# Digital Forensics for Malware Classification: An Approach for Binary Code to Pixel Vector Transition

**DOI:** 10.1155/2022/6294058

**Published:** 2022-04-21

**Authors:** Muhammad Rehan Naeem, Rashid Amin, Sultan S. Alshamrani, Abdullah Alshehri

**Affiliations:** ^1^Department of Computer Science, University of Engineering and Technology Taxila, Taxila, Pakistan; ^2^Department of Information Technology College of Computer and Information Technology, Taif University, P.O. Box 11099, Taif 21944, Saudi Arabia; ^3^Department of Information Technology, Al Baha University, Al Baha, Saudi Arabia

## Abstract

The most often reported danger to computer security is malware. Antivirus company AV-Test Institute reports that more than 5 million malware samples are created each day. A malware classification method is frequently required to prioritize these occurrences because security teams cannot address all of that malware at once. Malware's variety, volume, and sophistication are all growing at an alarming rate. Hackers and attackers routinely design systems that can automatically rearrange and encrypt their code to escape discovery. Traditional machine learning approaches, in which classifiers learn based on a hand-crafted feature vector, are ineffective for classifying malware. Recently, deep convolutional neural networks (CNNs) successfully identified and classified malware. To categorize malware, a smart system has been suggested in this research. A novel model of deep learning is introduced to categorize malware families and multiclassification. The malware file is converted to a grayscale picture, and the image is then classified using a convolutional neural network. To evaluate the performance of our technique, we used a Microsoft malware dataset of 10,000 samples with nine distinct classifications. The findings stood out among the deep learning models with 99.97% accuracy for nine malware types.

## 1. Introduction

Malware assaults increasingly pose a serious security threat to the internet and computer networks. According to Symantec research, 123 million devices record hundreds of harmful threat behaviors per second [1]. They are the most widely recognized computer security dangers. The figures are staggering, with some antivirus vendors reporting daily malware samples of more than 5 million. The number of internet-connected devices is predicted to reach 200 billion by 2020 [2], and they can be elaborate and add complexity, but you can be more specific. That malware is too much for security professionals to manage at once. To prioritize these events, a malware classification mechanism is frequently required.

Malware attacks on mobile devices and the internet of things (IoT) are becoming more common. Thanks to the complex system software environment and sensory devices, adversaries will find it easier to attack the system. Malware is harmful software that wreaks havoc on our digital systems' functionality, privacy, and dependability. There are several forms or families of malware, such as Trojans, Backdoors, and Worms, among others. Viruses and malware are currently among the most dangerous threats to our systems [3].

To conceal their identity, malware authors utilize a variety of approaches and strategies while writing code. As a result, determining the malware family or kind is the most difficult component. Traditional antivirus software struggles to keep up with the massive volume of malware that emerges every day. Computer scientists and antivirus companies have begun to use machine learning models to overcome this problem. Academic researchers and developers have proposed machine learning classifiers such as neural networks and logistic regression to classify malicious software [4, 5]. Clustering and categorizing the files' respective families are the first steps toward efficiently arranging and assessing many of them. Furthermore, such clustering criteria may be useful in detecting harmful and specific family members of newly found files on our computers. We require malware files with their relatives to group them and define new malware based on those clusters to enable study in this sector. As a result, the malware industry has evolved vast and is well organized. In the internet era, malware attacks on financial institutions and common individuals are rising. To deal with this fast malware development, flexible malware categorization algorithms for variants of malware files belonging to the same family are required [6–8]. A method of classifying malware by its family, regardless of whether it is a true variation, looks to be a very productive and successful technique for dealing with malware's fast expansion. Here are a few scenarios in which a solution to this challenge may be beneficial. Antimalware generator is the first, while malware developer identification is the second.

Analysts benefit from malware classification since they can further probe the malware's operations. Malware with similar structures is clustered together into a single cluster [9]. Furthermore, by identifying the malware's family, we may understand how it operates. Because of high-tech development evolutions in the digital software environment, mobile environment, social networks, smart cities, cloud computing, internet of things (IoT), and other areas, malware analysis and classification are a fast-growing sector requiring attention. Researchers have achieved great results using recurrent neural networks (RNNs) for speech recognition and handwriting identification [5, 10]. Many researchers have used machine learning approaches to identify and categorize malware. Machine learning-based malware detection comprises two phases: the first involves extracting features from photographs, and the second involves malware categorization [11, 12]. Using theoretical methodologies, automated malware detection takes too long and produces inaccurate findings. The automatic program that implements the new systems learned from this manual study improves its performance. We were inspired by Fred R. Barnard's quote, “A picture is worth a thousand words.” This study also looks into malware to determine whether the same holds. Visualizations have always been important in gaining a clear understanding of any framework or data. Visuals make more sense to us logically than any other representation [13, 14]. So the issue now is whether these sequential models can effectively detect malware families. Deep learning models were utilized in our research to categorize malware into different families.

Most malware classification algorithms employ feature vectors, which indicate harmful traits [15]. This research will classify both existing malware and new malware generated in the future. Antimalware businesses can swiftly develop antimalware for current malware and any future malware. Our main contribution to this research is that we use DL models to classify malware of various forms, and we examined previous work on malware image-based classification. Our methodology does not need feature engineering or domain expertise, such as binary disassembly, reverse engineering, or assembly language. Because we have supplied the picture dataset straight to the model as input, our technique may be used for real-time classification due to the low preparation time. We tested our model against the Microsoft dataset to categorize the new malware, which contained nine different malware families. We created a novel malware classification method that is computationally cost-effective, scalable, and efficient, based on single or hybrid deep learning models.

## 2. Related Work

More than a few researchers focused on malware visualization to better classify and identify malware studies to get maximum accuracy and in less time. This research section presents the related malware identification, visualization, and classification for which we used deep learning and ML models as a foundation.

Shaid et al. [16] proposed an approach to malware detection that relies on the unique behavior of malware executable files, which has been presented. The main thing is to identify any similarity in the conduct of malware samples. The researchers executed the malware executable file in the virtual environment in this technique. When they run the malware, something like an image or pattern is generated. They check the pattern sequence using a color map and check the similarity of the behavior in those images by using some statistical techniques. They got higher accuracy ranging from 95.91% to 98% by taking 1,102 malware image samples, and they got it from 12 different malware families. But this approach of exception malware in a virtual environment proves very time-intensive. Naeem et al. [17] introduced a malware classifier that worked on feature extraction first, and then to categorize the virus, they adopted a support-vector machine (SVM). This technique got 97.4% classification accuracy using a dataset containing 25 malware families with 9,339 sample files. But as a result, conventional methods need a feature analysis that takes a long time to compute.

For image categorization, we turn to deep learning to solve this problem. Our proposed solution is based on a new methodology recently developed. The author applies CNN for malware [5, 18]. Their base model applied different architectures, but the model is relatively narrow in that working style. Jhu-Sin Luo proposed the method that executes with GPU by using TensorFlow, which takes a significantly shorter time for processing. But, this scheme did not work on the virtual device and cannot identify the malware behavior. This technique is based on image recognition. But there are still some flaws in this method. If a developer differently rewrites the complete code, the results will be changed, and this technique will fail [11]. Dey et al. provide information on existing malware detection techniques based on image processing. They used an entropy filter to find the patterns in the images and got a better result [12, 19] than Natraj et al. [20]. Han et al. propose many techniques of visual matrixes shown in RGB colors based on the opcode sequences in the executable malware files; subsequently, to determine how similar the two datasets were, they were compared using entropy graphs between images. But these techniques worked only for the Windows PE files and were unable to work on packed data samples [21].

A new feature fusion technique to reassemble the features extracted from pretrained AlexNet has been proposed here. They used 25 classes to identify the malware and used the images removed from it to identify the malware using various SVM, decision tree, and K-nearest neighbor variants [13]. Utilizing these machine learning variants achieved 99.3 percent accuracy [10, 22]. Natraj et al. [20] introduced a different scheme of malware classification by feature extraction. The author converted the binary executable malware file into 2D grayscale images. They generated images and used a visible pattern or features to detect malware. Results indicated that it was more accurate and substantially less time-consuming than previous methods. To strengthen the security of existing infrastructure, scientists look at malware samples to figure out how they operate and the tactics that malware authors utilize. Malware analysis is being used for the classification of malware [7]. Determining which class a piece of malware belongs to is known as classification. After determining that a file is malware, we must evaluate its family. Static and dynamic malware analyses are the two main forms of analysis. It is possible to analyze a sample without running it through a process known as static analysis. In contrast, dynamic analysis is the process of executing a sample to determine its behavior like how it performed in different environments [23]. But we used a different approach; we have analyzed malware files by converting executable malware files into grayscale images, so in this way, there is no harm to our system, which does not need to execute the file.

### 2.1. Static Malware Analysis

Static malware analysis involves thoroughly testing a binary without attempting to execute it. This approach can be used for a variety of executable representations. It is much easier to perform static analysis when the actual code is present [17]. The binary file can be disassembled, and the assembly code is examined if the code is not accessible. Printable strings in the program's header, deconstructing the program, identifying byte sequences, examining the file's structure, and so on are all examples of static analysis [24, 25].

### 2.2. Dynamic Malware Analysis

It is possible to identify malware via dynamic analysis by running an executable file and observing its behavior. The malware is operated in a virtualized environment or VM to keep the virus' effects contained. Using a virtual machine, we can take a snapshot of the system before the virus starts running, and we can quickly revert to the saved state once the analysis is complete [26].

Images depicting malware assaults, such as spear-phishing attacks, were used in another study to describe the timeline of the attack, with colors indicating which sorts of system connections were successful [27]. However, applying only one feature is insufficient for effective real-world malware detection or classification context since malware writers' obfuscation tactics may obscure a feature utilized in the machine learning model. Therefore, there is a need to develop algorithms to deal with a wide range of traits. Current techniques can be split into two groups depending on where the features are merged. Early or data-level fusion techniques merge many data sources into a single feature vector, subsequently fed into a machine learning algorithm [28, 29]. As n-gram systems need human counting of n-grams during training, a convolutional neural network-based technique reduces this need. N-gram-like signatures are alternatively learned using convolution. This approach avoids the conventional feature extraction pipeline, feature selection, reduction, and classification because both algorithms are immediately tweaked during training [30].

Despite the rising danger posed by Android malware, researchers still lack a comprehensive understanding of common behaviors and developing patterns across malware families operating on the platform. Without this method, researchers risk developing algorithms that identify just historical threats while ignoring the most current ones. The author [31] analyzed approximately 1.2 million malware samples from 1.28 K families over eight years, making it the most comprehensive research of Android malware behavior. The author's objective is to understand better how Android malware has grown, with a particular emphasis on malware repackaging. Many harmless programs are piggybacked with a malicious payload (rider) in this sort of attack, enabling low-cost malware manufacture. Slicing the software to determine which components are benign and malicious is one of the most time-consuming aspects of examining repackaged malware. The author employed differential analysis to isolate irrelevant software components from the campaign to overcome this issue, enabling him to focus only on the bad riders' behavior. The analytical approach is based on publicly available data repositories and recent improvements in the systematization of antivirus information obtained from several sources. According to the author, the Android malware ecosystem has significantly evolved since its inception in 2010, regarding the kind of destructive activities carried out by malware and the amount of obfuscation utilized to escape detection. Finally, the ramifications of the results are discussed for Android malware detection research, emphasizing areas where the research community should concentrate its efforts. The ridership of malware families, in particular, varies with time. This reveals a substantial experimental bias in research that use automated algorithms to identify families without accounting for variance.

While fast expansion indicates an ecosystem's health, it creates challenges for mobile software developers in terms of generating and maintaining high-quality products and customers are worried about the usability and security of emerging apps. In this context, it is vital to give valuable and practical tools' help to mobile software developers that are informed and enabled by a full understanding of the ecosystem's evolutionary processes. The author [32] seeks to develop an architecture capable of systematically and continuously mining a mobile software ecosystem, emphasizing Android. Large-scale ecological longitudinal characterization research is conducted using this platform. To understand the ecosystem's evolutionary dynamics, the focus should be on the behavioral development patterns of ecosystem components such as mobile platforms, user applications developed on the platforms, and app users. Additionally, the characterization findings enable proactive app quality assurance and long-term app security. Additionally, the author examines risks and future steps and provides an update on this project with early data.

Given the frequent changes to the Android framework and the continued growth of Android malware, it is difficult to detect malware in a scalable and efficient manner over time. To address this issue, the author [33] presents DroidEvolver, an Android malware detection system that can automatically and continuously self-update while detecting malware without human intervention. While most existing malware detection systems can be updated by retraining on new applications with true labels, DroidEvolver can be updated without retraining or true labels, due to the insight that DroidEvolver updates itself via evolving feature sets and pseudolabels via online learning techniques. DroidEvolver's detection performance was examined using a dataset of 33,294 benign and 34,722 malicious apps created during six years. The F-measure of DroidEvolver is on average 2.19 times better than that of MAMADROID's state-of-the-art overtime malware detection system MAMADROID, and DroidEvolver's malware detection efficacy is 28.58 times more than that of MAMADROID. Additionally, DroidEvolver is immune to code obfuscation methods that are widely used.

Current malware detection methods for Android are dominated by machine learning-based categorization. On the other hand, current techniques are significantly constrained by their dependence on fresh malware samples that may not be instantly accessible and on ongoing retraining, which may be rather costly given the rapid growth of both the Android platform and its user apps. As a result, new and developing malware make their way through, as seen by the ongoing growth of malware in the wild. As a result, a more practical detector must be accurate on a subset of datasets and preserve its capabilities over time without needing retraining. The author [34] presents and investigates the sustainability issue for learning-based app classifiers in this study. This study establishes sustainability indicators for five cutting-edge Android malware detectors.

Additionally, the author created DroidSpan, a ground-breaking categorization technique for Android apps based on a unique behavioral profile that captures sensitive access distribution through lightweight profiling. The author compared the endurance of DroidSpan to five baseline detectors over eight years using longitudinal datasets. There were 13,627 benign programs and 12,755 malicious programs in the datasets. DroidSpan exceeded all baselines in terms of sustainability at a reasonable price by 6%–32 % for same-period detection and 21%–37 % for overtime detection, according to rigorous testing. The important takeaway, which also explains DroidSpan's success, is that learning-based malware detection requires the usage of persistent features that distinguish malware from benign apps over time, which may be uncovered via an app evolution study.

Machine learning techniques for Android malware detection must be periodically refreshed; otherwise, the trained classifier may be unable to distinguish newly found or developing malware kinds. This project aims to create a long-term Android malware detector that, once trained on a dataset, can detect new infections without requiring retraining. The author [35] examines how benign and malicious application behaviors evolve and determines the behavioral characteristics that consistently identify benign and malicious apps. The first results show that this approach has a promising future. On a seven-year benchmark, the proposed technique achieved exceptionally competitive detection accuracy for up to five years, outperforming the state-of-the-art, which lasted just two years.

Classification based on machine learning has long been a prominent way of malware protection. While there are many learning-based malware detection systems for Android, malicious applications continue to arise with increasing frequency in different Android app marketplaces. “How it is that new and developing malware can evade such a broad range of detection techniques?” the author [36] inquires in this regard. Intuitively, the performance deterioration of malware detectors may be the core reason for their failure to identify new infections after training on older samples. This research examines the degraded performance of four cutting-edge Android malware detectors to address the problem. The author verified that these present solutions significantly deteriorate and fast over time. The author introduces a unique categorization approach based on a long-term characterization study of Android apps focusing on dynamic behaviors. It compared our innovative technology to four current detectors and discovered considerable advantages for our new system. The key point is that studying app development over time may aid in detecting malware.

### 2.3. The Evolution in Malware Classification

Other systems had malware before 1986, but the PC had the first. Brain.A was the virus. Basit and Amjad, two Pakistani brothers, created it. They constructed a virus that replicated using floppy discs to demonstrate the PC's vulnerability. It infected the floppy drive's boot sector and every inserted floppy disc. One is the Omega virus. It was dubbed Omega because of the omega symbol engraved on the console. The Michelangelo virus rewrote the first 100 hard drive sectors in 1992. Walker, the next virus, arrived in 1992. It was an animated walker crossing the screen. The ambulance virus, like Walker, animated an ambulance vehicle traveling across the screen, but it also included sound effects [37, 38]. The Casino virus was one of the most intriguing viruses of the early 1990s. The Casino virus copies the file allocation table to memory and deletes the original. Then, it offers the user a slot game.

The history of malware may be divided into five categories, each corresponding to a historical period during which events in that category occurred. The first is malware development in its early stages. The first malware began to arise during this period. The early Windows period is the second category, while the evolution of network worms is the third. With the extensive use of the internet, malware has grown more common [39–41]. Rootkit and ransomware fall under the fourth group. Before 2010, this was the most hazardous evolution of malware. Rootkit and ransomware constitute the fourth category. Before 2010, this was the most destructive evolution of malware. We look at malware designed for virtual spying and disruption.

**Figure 1:**
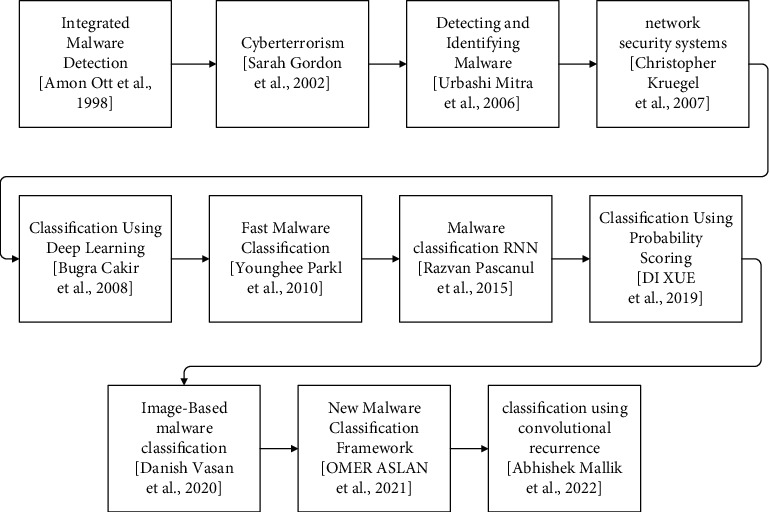
The evolution process in malware classifications.

Some nations' spy agencies generated this virus. This is the newest stage of malware development [42, 43]. Malware production evolved from showmanship, vengeance, and profit to espionage and sabotage. Profit is still a driving force behind malware development and will be in the future. Malware authors have used espionage and sabotage for military goals. It is safe for attackers to employ and may do the same damage as military assaults with all its might [7, 13]. It remains to be seen how antivirus businesses respond to attackers with practically infinite resources for malware production and profit-driven malware developers. When it comes to military usage of malware, we may see more events like Stuxnet in the future. It remains to be seen how antivirus firms will cope with attackers with almost unlimited resources to create malware and those motivated only by profit [44, 45]. However, with occurrences like Stuxnet, we may see alternative uses for malware in the future and malware classification evolution is shown in Figure 1.

## 3. Methodology

This section explains how the deep learning algorithm is used to classify malware. For the classification issue with several classes, we provide a novel approach. The malware executable is first converted into grayscale graphics using our suggested technique. The photographs are fed into a fine-tuned deep learning model to identify and categorize the malware family. So, by placing the malware family, we get an idea about malware behaviors and types. In this way, it will be helpful for malware analysts to search for that specific behavior and generate antimalware. It is challenging to propose a wide-ranging malware classification system that can handle a massive quantity of malicious code and identify its family. In this section of the methodology, we discuss the steps that we performed to do this classification; the steps are as follows: data collection and preprocessing of the dataset, visualization of binary to a grayscale image, mode training, and model testing.

### 3.1. Data Acquisition

Microsoft is giving an unprecedented malware dataset to the data science community and promoting open-source progress on successful approaches for categorizing malware files into various families. Microsoft provided known malware files from nine distinct families that are included in this collection. Id, a 20-character hash value, and Class, a number, designate one of nine family names to which the malware belongs. Remnit, Lollipop, Kelihos ver3, Vundo, Simda, Tracur, Kelihos ver1, Obfuscator.ACYm, and Gatak are some of the several malware families.

### 3.2. Preprocessing of Data

Each file's binary information, excluding the PE header, is represented in hexadecimal form in raw data format. It is also possible to get a list of all metadata information collected from the binary, such as function calls and text values. The IDA disassembler was used to produce this. Malware dataset contains the following files.

  Training File: - This file contains the raw data for the training set (MD5 hash = 4fedb0899fc2210a6c843889a70952ed).
  Testing File: -This file contains the raw data for the test set (MD5 hash = 84b6fbfb9df3c461ed2cbbfa371ffb43).

### 3.3. Labeling

We need labeled samples because we are implementing supervised learning for classification. So, for marking these samples, we used the Microsoft Official dataset provided by Kaggle for competition. We have to supply the binary's MD5 hash for this.

TrainLabels.csv—the training set's class designations.

### 3.4. File Separation Process

**Figure 2:**
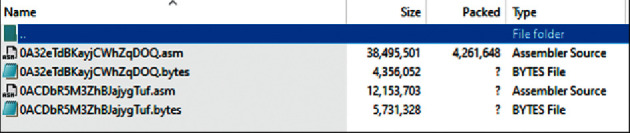
Original dataset file having both byte and .asm files.

**Figure 3:**
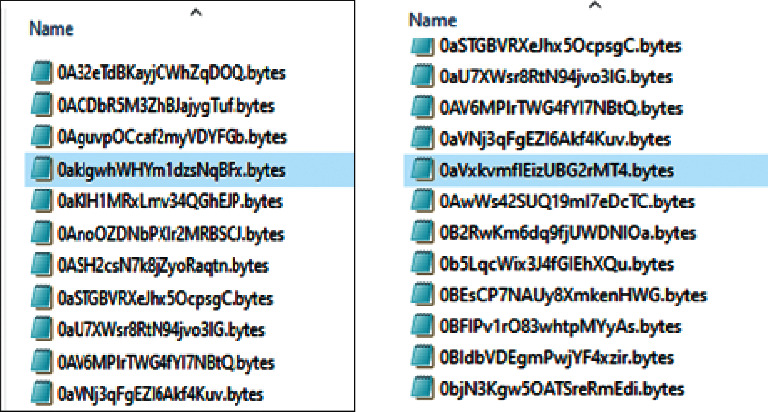
Byte files are taken from the dataset. Each file shows a different identifier, and it has its class label.

In the dataset, we have the number of files having .asm as shown in Figure 2 and byte types as shown in Figure 3. We need to separate these files, so we use python language and PyCharm tool to code for this, and via code, we can split the byte and.asm files. We need the byte files for future use.

### 3.5. Byte to Image Conversion

**Figure 4:**
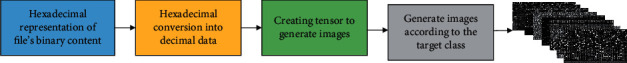
The image conversion process from hexadecimal binary content to 3-channel images.

Bytes files are included in our dataset. The raw data for each file provide the file's hexadecimal representation sans the file's PE header. So, initially, we transformed each hexadecimal representation into its decimal equivalent. There are 8-bit integers stored in a byte file. This one-dimensional vector can be easily turned into a two-dimensional array. An 8-bit grayscale picture with each pixel spanning from 0 to 8 may be easily viewed from 0 (black) to 255 (white) as shown in Figure 4.

### 3.6. Normalization of Image

**Figure 5:**
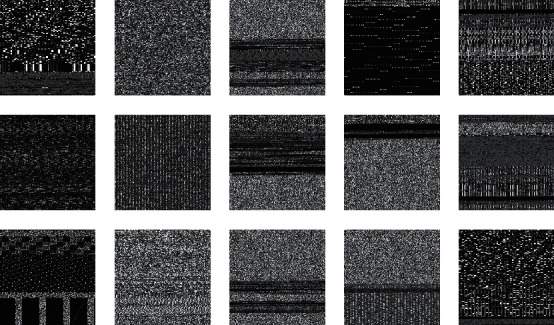
Malwares' families are represented in different image forms.

Preprocessing the data before they are sent to the network is normalization. They are created by malware binaries and have no predetermined dimensions, making them difficult to categorize. To solve this issue, we initially reduced the size of the virus images to 224 × 224 pixels. Thus, the malware pictures were standardized and prepared to enter the CNN as input information. The key advantages of the normalizing method were that it reduced the size of the input photographs and made them more suitable for network training. The dimensionality reduction process also omitted several important features. We found that most malware pictures in our collection maintained their texture after the normalization process. Here are some image representations of malware from different families as shown in Figure 5.

### 3.7. Feature Extraction

This stage is critical for classifying deep learning models with the needed features. When dealing with enormous amounts of data, it is sometimes necessary to reduce the data to a more manageable number of feature representations. Resizing the image is part of data preprocessing. Apart from that, features can be extracted after and before data preprocessing according to the system design. Images are created from the binary file to the byte file and in the images. The image feature set can be desired while working on image data. Image tensors store a chunk of images for model training and testing phases. A feature contains information on an image's dimensions, texture, color, and shape. Local, global, and textural characteristics were all utilized in this case. A data stream is used to extract features in two steps in the proposed framework. Its durability and cheap processing cost make it an excellent first-stage tool for extracting texture features from grayscale photographs. Using the pretrained CNNs, a robust classification model is generated by extracting additional deep features.

### 3.8. Implementation

**Figure 6:**
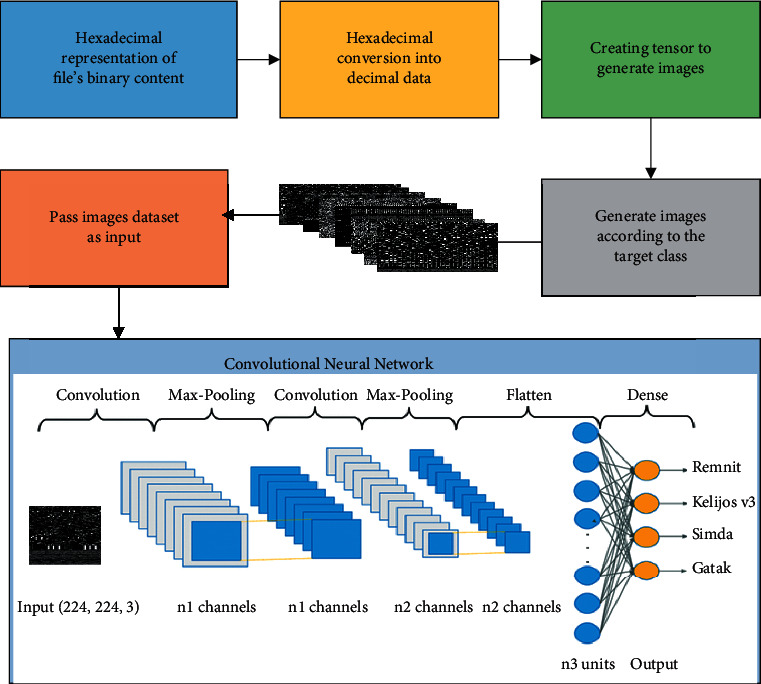
Proposed model.

We adopt a different approach to analyzing and classifying malware than previous approaches. To address this issue, we turn to a convolutional neural network (CNN), a machine learning architecture that uses deep learning techniques, as shown in Figure 6. Deep learning has recently provided maximum efficiency across various applications and scenarios in numerous domains, including natural language processing, computer vision, speech recognition, and bioinformatics. However, using CNNs in many other fields has not been well investigated. Cyber security is one industry that could significantly benefit from developments in deep learning. With the recent success of deep understanding (particularly CNNs) in numerous classification tasks, we believe it can classify malware superior to support-vector machines in terms of accuracy. For image-processing issues, CNNs, in particular, have shown to be particularly successful.

For this reason, we turn malware classification into an image classification issue that can be tackled with CNNs. We design a generic malware classification architecture based on a deep convolutional neural network (CNN) instead of the current approaches. Existing high-accuracy approaches are frequently adapted to a given dataset. On the other hand, in the suggested process, the discriminative representation is directly learned from the data, rather than through hand-crafted feature descriptors, making it data independent. In the first step, raw data for each file provide the file's binary content sans the PE header in hexadecimal. So, initially, we transformed each hexadecimal representation into its decimal equivalent. Using each unit's top and lower nibbles as indices when creating a two-dimensional color map, we may generate a series of RGB (pixel value) values for each hexadecimal digit. Image representations were then created by concatenating this sequence of pixel values to produce a two-dimensional matrix. A dataset containing photographs of malware is obtained in this way. For each image, it is adjusted to fit 224 rows of columns. A total of 10,000 samples were utilized for training and validation from a dataset made up of data from nine different classes. A twelve-layer residual network processes the training set samples. The model comprises two layers of convolution, followed by a layer of max pooling, followed by two layers of convolution, a layer of max pooling, and so on by adding flattened and dense layers.

## 4. Results and Discussion

Experimental results are shown in this section of the article. Texture-based malware classification is resistant to obfuscation methods and to enhance accuracy, according to the literature. Over 80% of malware binaries adopt this technique. The model is evaluated using a Microsoft Kaggle malware dataset, which was further converted into 224 × 224-pixel grayscale images from byte files using a deep convolutional neural network (CNN) approach. There were 100 epochs of training and testing with 9 classes of images with 10,000 samples, and our proposed model's accuracy was 99.97%. There are nine distinct malware programs in our dataset; however, the suggested study still outperforms them all.

### 4.1. Evaluation Criterion

The experiments were implemented in Colab and Kaggle 64 bits on computer servers having 16 core CPU Xeon processors each of 3.2 GHz. 32 GB of RAM and 8 GB GPU. For the validation, an evaluation process of the implemented model testing process for the model is executed. The preliminary performance evaluation matrixes are the time and integrity of the predicted data. We will show the model training and testing evaluation based on accuracy, precision, and recall for both training and testing.

**Figure 7:**
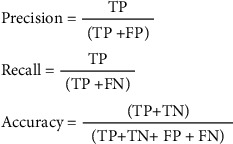
Formulas of precision, recall, and accuracy.

The performance of the prior system model is compared to our new, improved system scheme and machine learning model. The criteria listed below are used to assess the accuracy and other performance characteristics, as shown in Figure 7, where TP means true positive, FP means false positive, TN means true negative, and FN means false negative.

**Figure 8:**
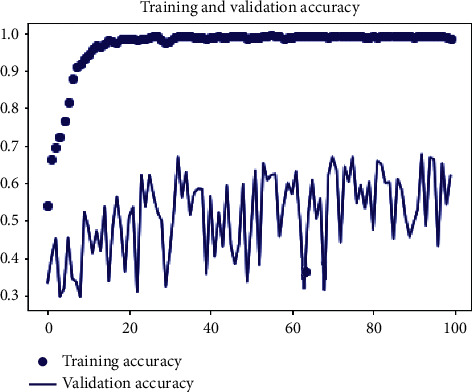
Training and validation accuracy.

There were 100 epochs for the dataset that was used for training and testing.  Figure 8 demonstrates the training and validation accuracy values, which are 99.97% as shown in the graph. The validity accuracy value grew in our model while the training loss value declined. In fact, depending on how some parameters are designed, different models may be able to increase verification capabilities. On the other hand, we have been unable to validate alternative models using Microsoft's dataset in our method.

**Figure 9:**
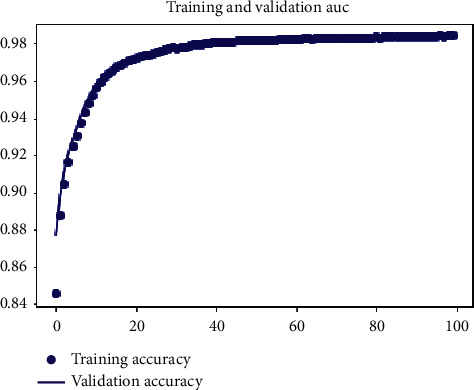
Training and validation of AUC.

Figure 8 depicts the validation performance results, and it shows that training and validation are almost identical curves for our model.  Figure 9 shows that the former had a greater accuracy and a lower loss since it had a bigger number of samples. This finding demonstrates that severely lowering sample data by undersampling affects classification accuracy.

**Figure 10:**
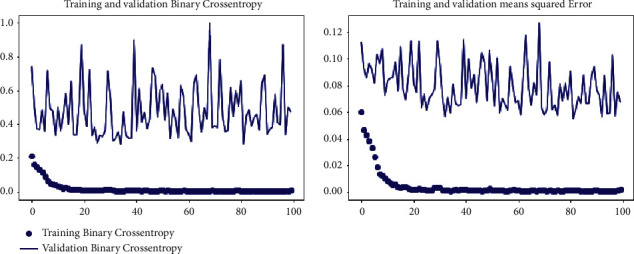
Training and validation binary cross-entropy and training and validation mean squared error.

Figure 10 describes the binary cross-entropy results as shown in the below graph. After training the model on binary cross-entropy on the dataset, we validated the model, showing 99.97% results. Training and validation mean squared error is showing that our model performance is good on all 100 epochs.

**Figure 11:**
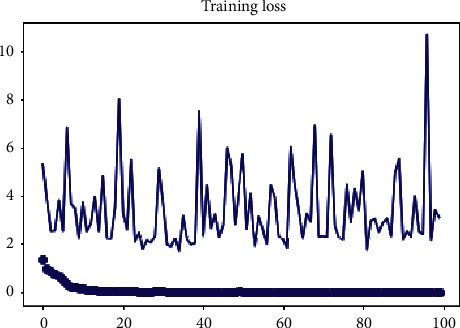
Training loss.

Training loss is very small in our model, which works fine. In Figure 11, training loss is very small and validation is high, which evaluates our proposed model's accuracy and performance. The results of the experiments confirmed that our suggested technique is resistant to polymorphic obfuscation and that texture-based malware detection works.

Table 1 provides the model's statistical values predicted during the evaluation process. Our proposed model achieved an accuracy value, which is 99.97% as compared to the other state-of-the-art methods.

## 5. Conclusions and Future Work

This manuscript explains the malware detection process by using deep learning techniques. Our proposed methods show the highest accuracy values. The accuracy comparison of the proposed model is the highest one, i.e., 99.97% compared to the already existing algorithms. In our model's simulation, the validity accuracy value increased, but the training loss value decreased. Because of this, various models may be able to improve their ability to verify the information. On the other hand, we have been unable to validate alternative models using the Microsoft dataset in our method. Even though we have seen some experimental proof of the recommended approach's effectiveness, more research in the following directions is required. We will improve a method for detecting and categorizing malware using GPUs and other parallelization methods to achieve high-performance computing. In the future, the large-scale application situations are implemented, the suggested approach. More research is needed to efficiently detect malware with antidissembling, antidebugging, and antipacking methods. We intend to investigate the fundamental reasons for the deteriorating problem in the future and build more effective malware detection tools.

## Figures and Tables

**Table 1:** Validation of new algorithms against the Microsoft malware dataset.

Method	Epochs	Dataset	Accuracy
Vinayakumar et al. [46]	100	Microsoft malware dataset	91.27
Cui et al. [47]	10	Microsoft malware dataset	93.4
Luo and Lo [11]	60	Microsoft malware dataset	93.57
Singh et al. [48]		Microsoft malware dataset	94.24
Gilbert [49]	25	Microsoft malware dataset	94.64
Aslan et al. [44]		Microsoft malware dataset	94.88
Proposed method	100	Microsoft malware dataset	99.97

## Data Availability

The data used to support the findings of this study are available from the corresponding author upon request.
